# Supplementary material for: P2Y2R-Mediated PAK1 Activation Is Involved in ESM-1 Overexpression in RT-R-MDA-MB-231 through FoxO1 Regulation
Source: Cancers (Basel). 2022 Aug 26;14(17):4124. doi: 10.3390/cancers14174124 (PMC9454712; doi:10.3390/cancers14174124)
Supplement: Supplementary file 1 [file cancers-14-04124-s001.zip › Supplementary Materials-R1.pdf]

Supplemantary Materials

$P2Y_2R$ mediated PAK1 activation is involved in the ESM-1 overexpression in RT-R-MDA-MB-231 through FoxO1 regulation.

Hana Jin, Hye Jung Kim*

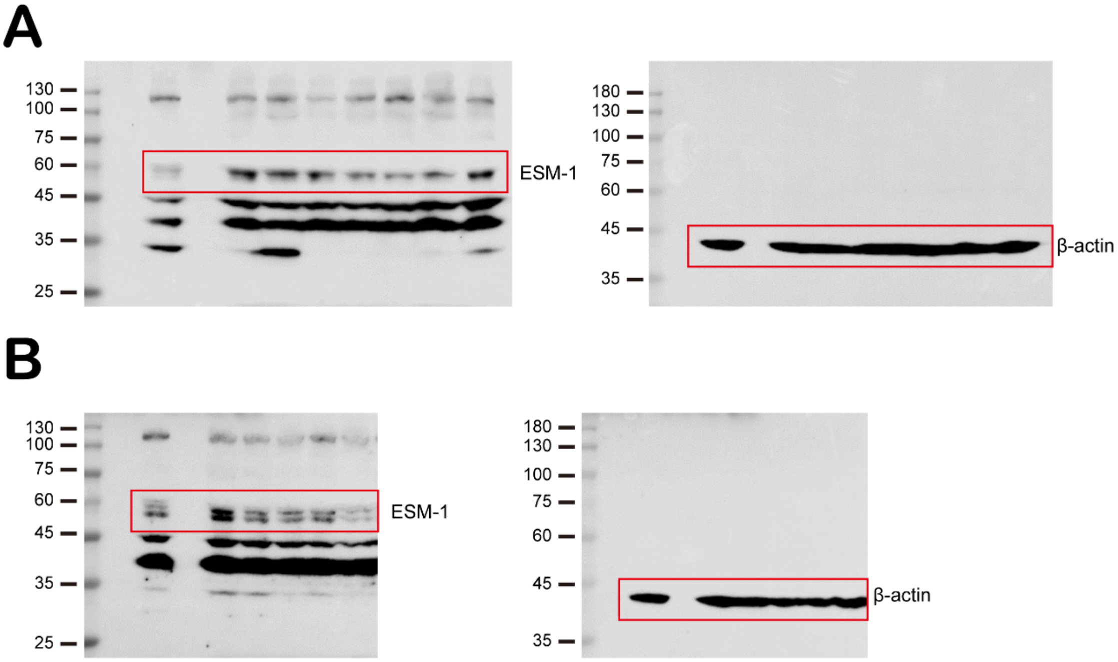

Figure S1. The original western blot images for Figure 1. Images in Figure 1C were cropped from (A) and images in Figure 1E were cropped from (B).

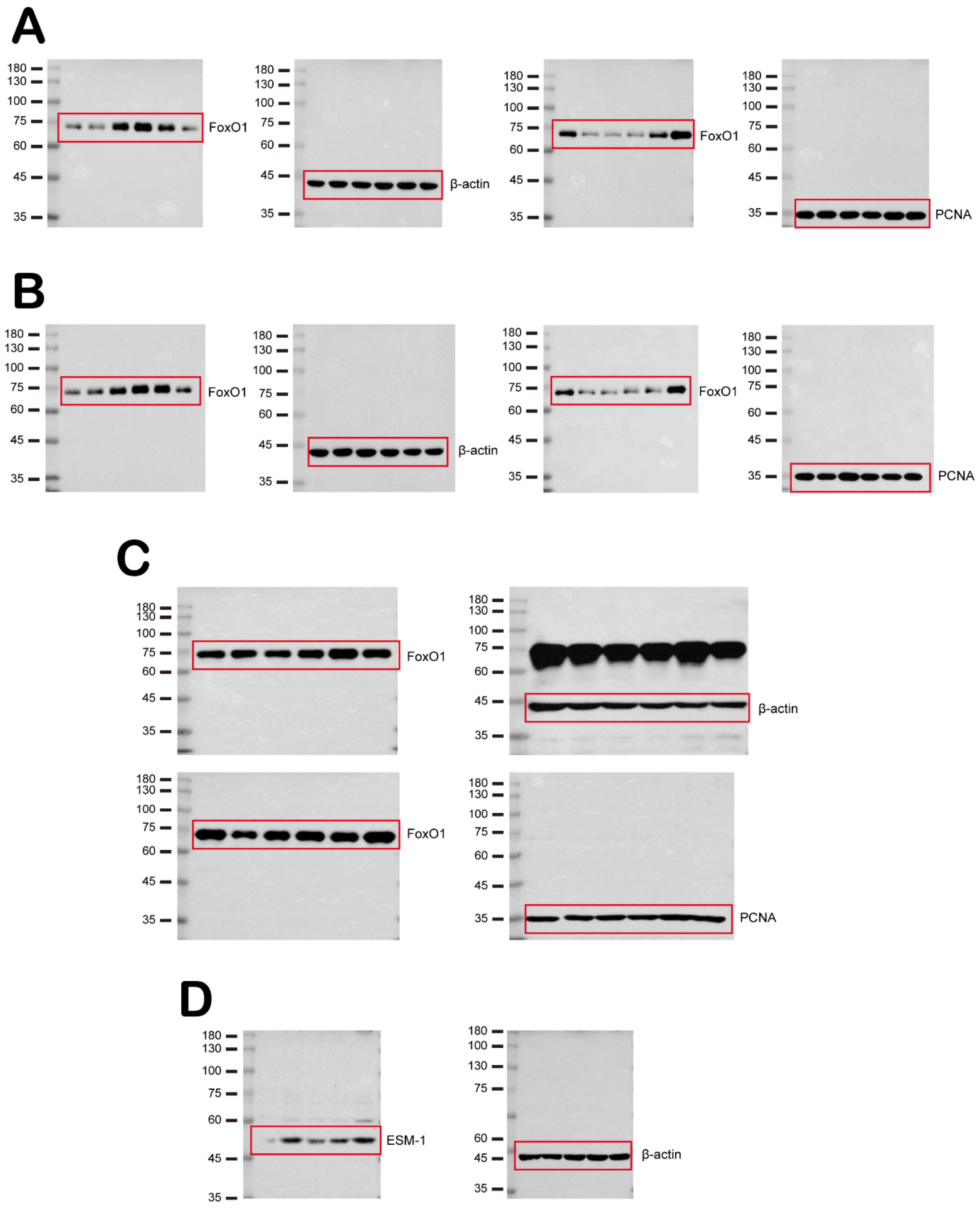

Figure S2. The original western blot images for Figure 2. Images in Figure 2A were cropped from (A) and images in Figure 2B were cropped from (B). Images in Figure 2C were cropped from (C) and images in Figure 2E were cropped from (D).

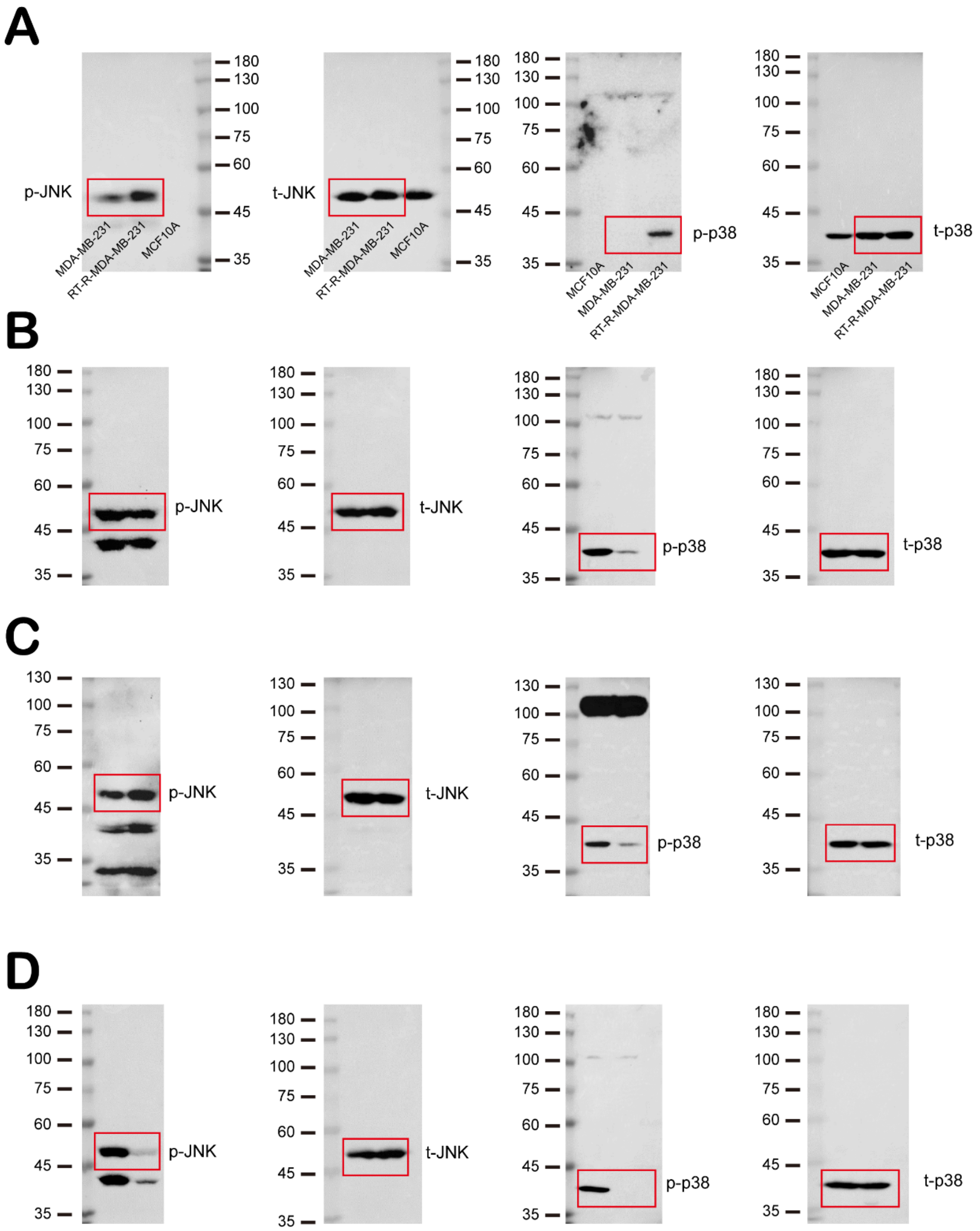

Figure S3. The original western blot images for Figure 3. Images in Figure 3A were cropped from (A) and images in Figure 3B were cropped from (B). Images in Figure 3C were cropped from (C) and images in Figure 3D were cropped from (D).

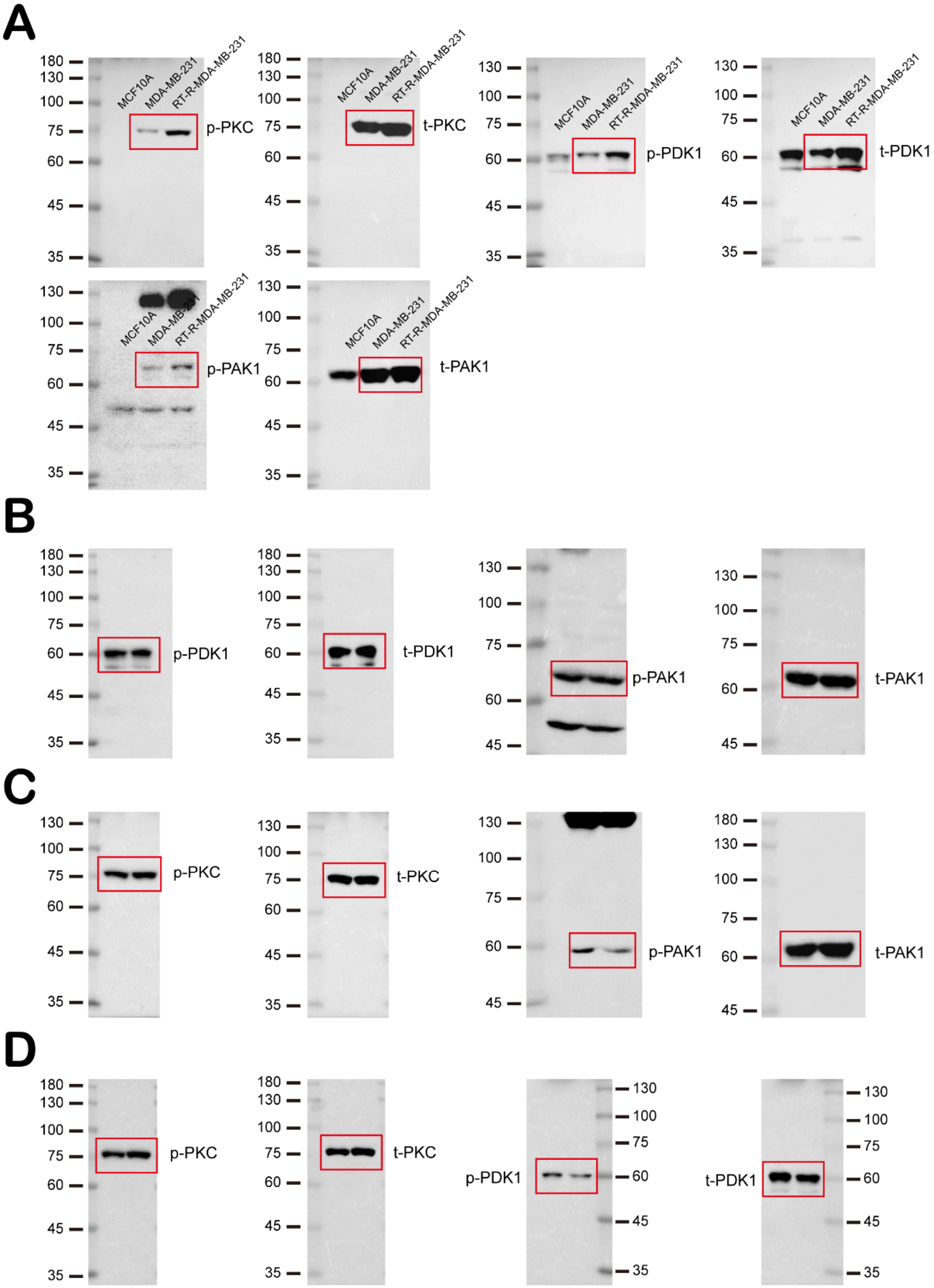

Figure S4. The original western blot images for Figure 4. Images in Figure 4A were cropped from (A) and images in Figure 4B were cropped from (B). Images in Figure 4C were cropped from (C) and images in Figure 4D were cropped from (D).

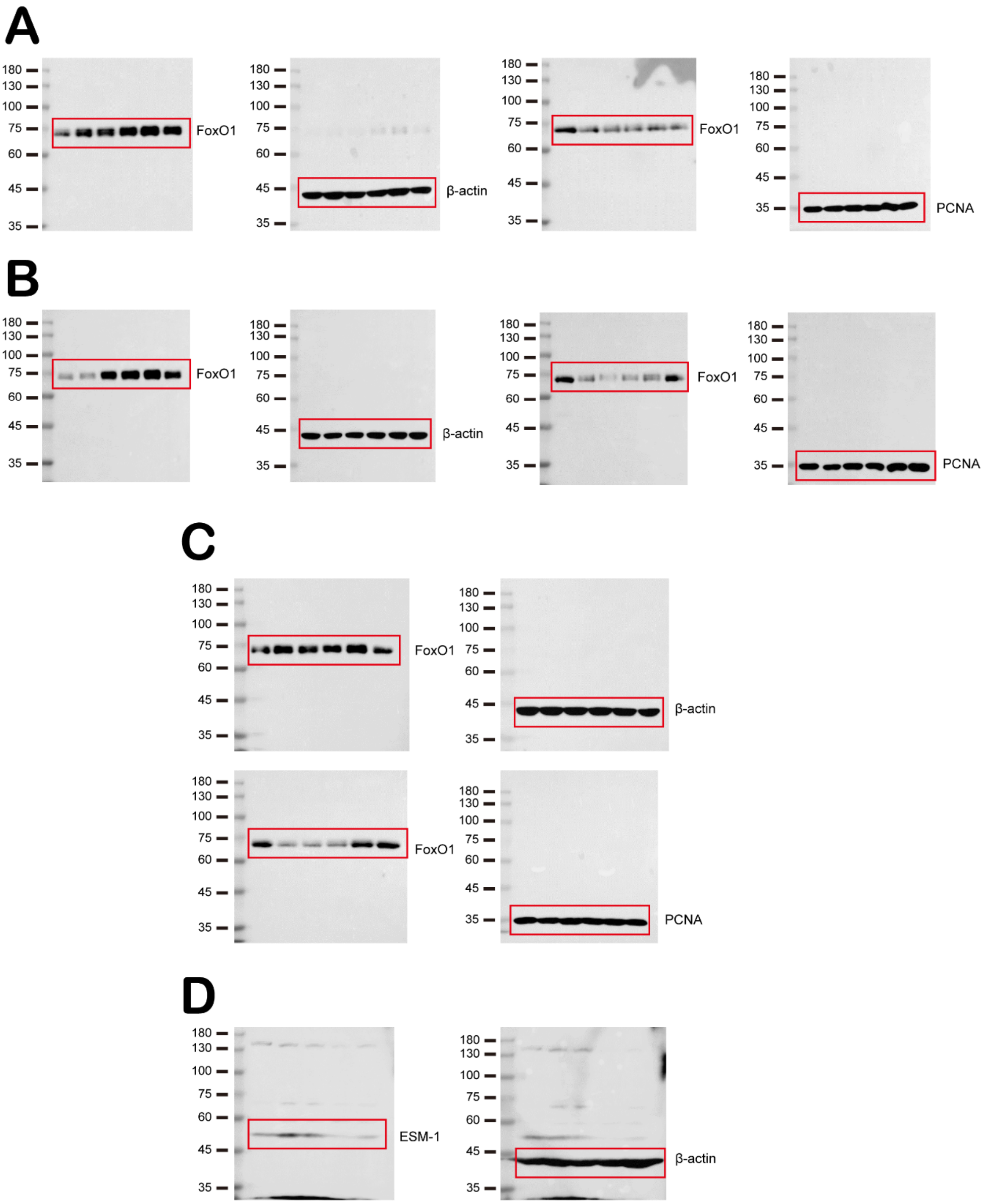

Figure S5. The original western blot images for Figure 5. Images in Figure 5A were cropped from (A) and images in Figure 5B were cropped from (B). Images in Figure 5C were cropped from (C) and images in Figure 5E were cropped from (D).

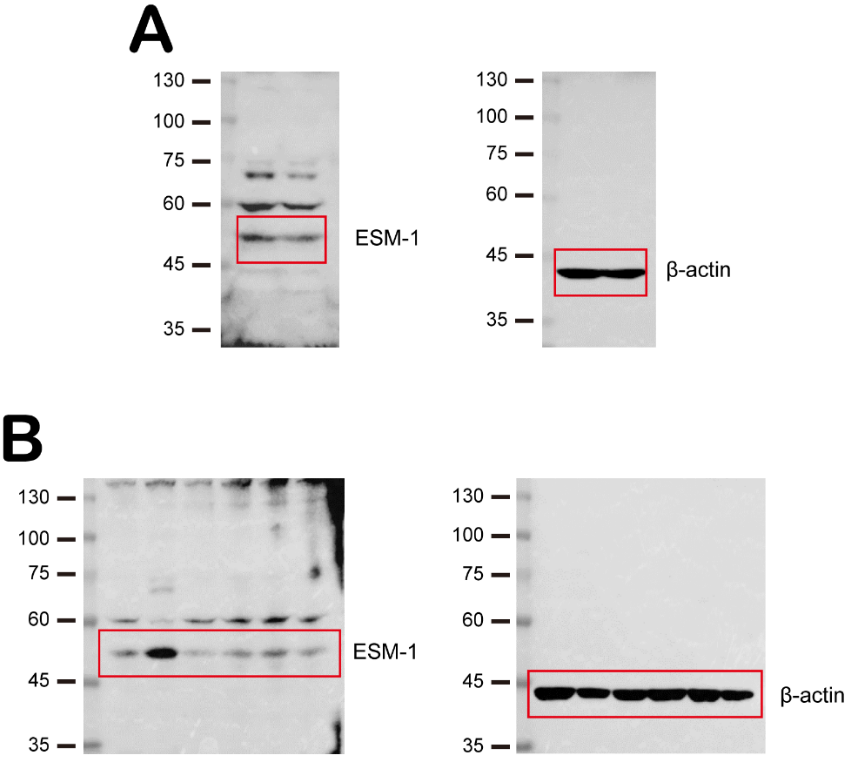

Figure S6. The original western blot images for Figure 6. Images in Figure 6A were cropped from (A) and images in Figure 6C were cropped from (B).

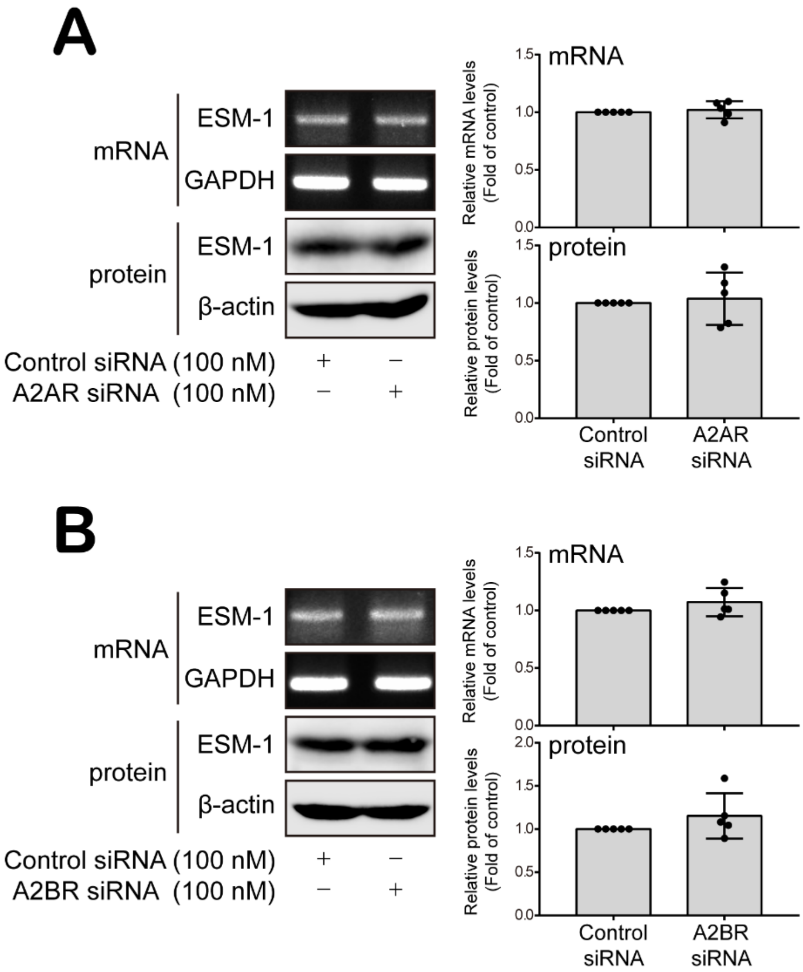

Figure S7. A2AR and A2BR siRNA did not affect ESM-1 expression levels. RT-R-MDA-MB-231 cells were transfected with negative control siRNA (control siRNA), A2AR or A2BR siRNA as described in the Methods section. Then, ESM-1 mRNA and protein expression levels were confirmed by RT-PCR (A) and western blotting (B), respectively. GAPDH and β-actin were used as loading controls. The values are presented as the mean ± SD of five independent experiments.

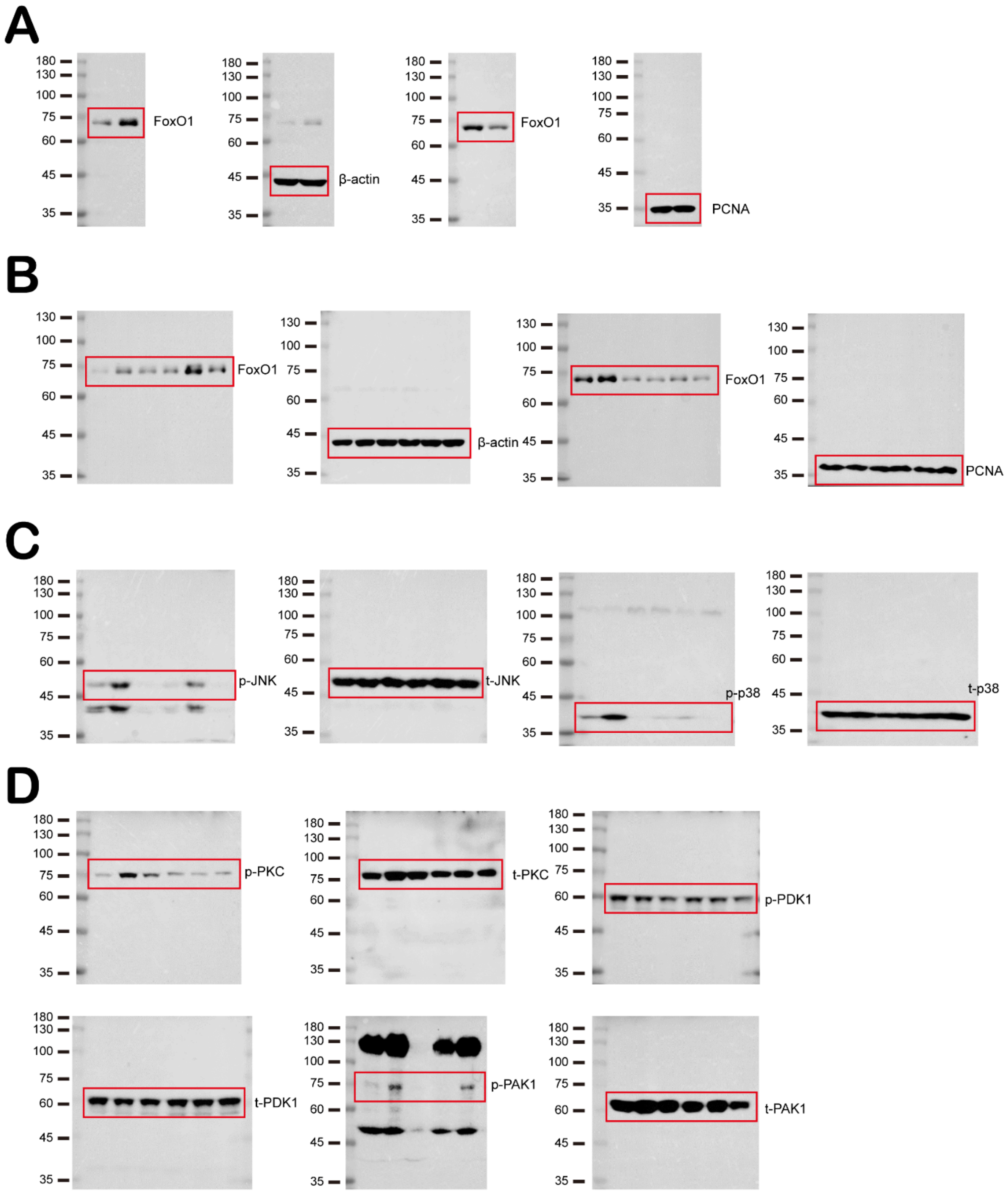

Figure S8. The original western blot images for Figure 7. Images in Figure 7A were cropped from (A) and images in Figure 7B were cropped from (B). Images in Figure 7C were cropped from (C) and images in Figure 7D were cropped from (D).
